# The Future of Pre-Exposure Prophylaxis (PrEP) for HIV Prevention: A Global Qualitative Consultation on Provider Perspectives on New Products and Differentiated Service Delivery

**DOI:** 10.1007/s10461-023-04093-1

**Published:** 2023-06-23

**Authors:** Mary Henderson, Heather-Marie A Schmidt, Lastone Chitembo, Hortencia Peralta, Ahmed S Alaama, Cheryl Johnson, Rachel Baggaley, Robin Schaefer

**Affiliations:** 1 Independent Consultant, Riverside, USA; 2 Global HIV, Hepatitis and STIs Programmes, World Health Organization, Avenue Appia 20, Geneva, 1211 Switzerland; 3 UNAIDS Regional Office for Asia and the Pacific, Bangkok, Thailand; 4 World Health Organization, Lusaka, Zambia; 5 Pan American Health Organization, Washington, DC USA; 6 World Health Organization Office for the Eastern Mediterranean Region, Cairo, Egypt

**Keywords:** HIV, Pre-exposure prophylaxis, Healthcare providers, Health services

## Abstract

Differentiated service delivery and new products, such as long-acting injectable cabotegravir (CAB-LA) and the dapivirine vaginal ring (DVR), could increase uptake and use of pre-exposure prophylaxis (PrEP) for HIV prevention. We explored PrEP provider perspectives on differentiated PrEP service delivery and new PrEP products to inform World Health Organization (WHO) guidelines and programme implementation. 150 PrEP providers who participated in a WHO survey were randomly selected and 67 were invited for interviews based on geographic representation, provider cadre, gender, experience with community-based PrEP service delivery, and familiarity with new PrEP products. Semi-structured interviews were conducted virtually. Key themes were inductively extracted relating to differentiated service delivery and benefits and concerns regarding new PrEP products. 30 PrEP providers from 24 countries were interviewed. Across regions, providers were supportive of differentiated service delivery to respond to clients’ needs and preferences, maintain services during COVID-19, and ensure access for priority populations that may face access challenges. Providers welcomed prospects of offering CAB-LA to their clients but had concerns about HIV testing, costs, and the need for clinic-based services, including staff who can administer injections. Providers felt the DVR was potentially important for some cisgender women, especially young clients and female sex workers, and raised fewer concerns compared to injectable PrEP. Providers’ views are critical for the development of guidelines and implementing programmes that will best serve PrEP users. Understanding areas where provider capacities and biases may create barriers can define opportunities for training and support to ensure that providers can deliver effective programmes.

**Supplementary Information:**

The online version contains supplementary material available at 10.1007/s10461-023-04093-1.

## Introduction

Since the World Health Organization (WHO) recommended offering tenofovir-based oral pre-exposure prophylaxis (PrEP) for HIV prevention to all people at substantial risk of HIV infection in 2015, there have been considerable changes in PrEP service delivery and use globally, although the availability, accessibility, and coverage of PrEP varies widely across and within countries and PrEP programmes commonly remain small-scale [1]. WHO PrEP recommendations have been widely adopted into country guidelines [1] and numbers of PrEP users globally increased to more than 1.6 million in 2021, although this is well short of the global target of 10 million PrEP users by 2025 [2].

PrEP is increasingly delivered through differentiated services, a trend accelerated by the COVID-19 pandemic [3, 4]. Differentiated service delivery adapts services to the needs and preferences of the people who are interested in and could benefit from PrEP. WHO has published guidance to support differentiated PrEP services [5], using a framework of four building blocks of differentiated service delivery: service location (where), frequency (when), package (what), and provider (who). Examples of service adaptations within those building blocks include community-, pharmacy-, and home-based PrEP delivery (“where”) [6, 7], multi-month dispensing to reduce follow-up visits (“when”) [8], integrated services (“what”) [9], and task sharing with various health worker cadres and lay providers (“who”) [10]. The use of HIV self-testing (HIVST) has supported many of these differentiated PrEP service delivery models.

In addition to changing models of service delivery, more PrEP products are becoming available. WHO now recommends offering the dapivirine vaginal ring (DVR) as an additional prevention choice for cisgender women at substantial risk of HIV (2021) [11] and long-acting injectable cabotegravir (CAB-LA) for all people at substantial HIV risk (2022) [12]. Offering a choice of PrEP products is another critical aspect of differentiated service delivery as it allows clients to choose the product that best suit their needs and preferences. This may make services more acceptable and thus improve uptake and effective use of PrEP, but implementation research is needed on how to effectively integrate new PrEP products, particularly CAB-LA, into PrEP programmes [13].

There is extensive literature focusing on the perspectives of PrEP clients and potential users that highlights diverse preferences for service delivery and PrEP products [14–16]. However, fewer studies have focused on the views of PrEP providers, and to date, studies focused on PrEP providers have largely been conducted in the US, with limited representation from low- and middle-income countries. Providers have important insights into the realities of their clients’ lives and potential barriers and facilitators to PrEP uptake and persistence, and they understand what is needed to effectively implement PrEP services. Providers may also pose barriers to successful programme implementation, for instance due to limited knowledge about service delivery innovations and new products [17–19] or biases and stigmatizing attitudes [20–24]. Therefore, to leverage the potential of differentiated PrEP services and new PrEP products in expanding PrEP access, uptake, and persistence, PrEP provider perspectives must be considered in guideline development, policy making, and programme planning and implementation. Understanding provider perspectives can help to inform strategies to strengthen PrEP workforce capacities and thereby further expand access to PrEP services and ensure delivery of comprehensive, non-judgmental, and effective services. This article describes the outcomes of a global consultation, involving qualitative in-depth interviews of PrEP providers, on providers’ perspectives concerning differentiated service delivery and new PrEP products. Results were utilized to inform WHO guidance and guidelines and to determine future provider training priorities.

## Methods

### Participants

From November to December 2021, an online survey was conducted by WHO among 1353 PrEP providers from 84 countries to gain insights into how PrEP services are delivered in practice. Respondents were asked if they would be willing to participate in a follow-up in-depth interview. Of the 556 survey respondents who agreed, 150 were randomly selected as possible interview participants using an online random number generator, and 67 of those were invited for interviews based on geographic representation, provider cadre, gender balance, experience with community-based PrEP service delivery, and familiarity with either CAB-LA or the DVR. Of the 67 who were invited, 43 did not respond and 24 agreed to participate and were scheduled for an interview. To ensure global geographic representation, an additional six PrEP providers were identified by technical staff of the WHO Global HIV, Hepatitis and Sexually Transmitted Infections (STIs) Programmes and were invited to an interview.

### Data Collection and Analysis

A semi-structured interview guide was developed in collaboration with the WHO Global HIV, Hepatitis and STIs Programmes (see the supplementary material for full guide). Interviews were tailored to each participant based on their online survey responses, with a focus on experiences with and perspectives on differentiated service delivery and new PrEP products. For new PrEP products, participants were asked about CAB-LA and DVR, including perceived benefits and challenges with the implementation of these products. For differentiated service delivery, questions were informed by the framework of the four building blocks of differentiated services (what, where, when, and who). Providers were also encouraged to suggest ways to improve access to and uptake of PrEP.

Interviews were conducted by the lead author (MH) in English via video calls between January and February 2022 and lasted 60–90 min. Where there were internet connectivity issues, participants were asked to provide answers to questions in writing after the interview. The interviews were digitally recorded and transcribed. Data were stored on a password-protected device. Recordings and interview notes were analyzed inductively to extract themes related to the building blocks of differentiated service delivery and anticipated benefits and concerns regarding new PrEP products.

### Ethics and Consent to Participate

The consultation described in this article was initiated as part of a stakeholder consultation to inform the development of WHO guidelines on CAB-LA. As such, it was carried out in accordance with the WHO Handbook for Guideline Development [25]. The development of all WHO guidelines is overseen by the WHO Guideline Review Committee. While the project was not set up as a formal research study, a strict protocol was followed according to which the purpose of the consultation was explained at the beginning of each interview, including that results may be published. Verbal informed consent was obtained before starting the consultation. All participants were informed that they could stop the interview at any point. When the decision to publish the results of the consultation was made, additional written consent for this publication was sought from participants.

## Results

### Participant Characteristics

Thirty interviews with 30 different providers were conducted (see Table 1 for geographic distribution). Participants included 17 physicians, eight nurses, one pharmacist, one researcher, one PrEP navigator, one counsellor, and one clinical psychologist. Thirteen participants identified as male, 16 as female, and one as non-binary. Four of the 30 interview participants provided additional written answers to clarifying questions due to connectivity challenges.

**Table 1:** Geographic distribution of pre-exposure prophylaxis (PrEP) providers who participated in consultations

Region^[a]^	Participants				Countries
	Total	Physicians	Nurses	Other^[b]^	
Africa	7	3	3	1	Mali, Namibia, Nigeria, Zambia, Zimbabwe, Uganda (2x)
Americas	8	4	2	2	Brazil, Dominican Republic, Guatemala^[c]^, Mexico, USA (4x)
Eastern Mediterranean	1	1	--	--	Lebanon
Europe	6	5	1	--	Spain, UK, Switzerland, Poland, Ukraine^[c]^ (2x)
South-East Asia	5	4	--	1	Sri Lanka, Thailand^[c]^, Myanmar, Nepal, Viet Nam^[c]^
Western Pacific	3	1	2	--	Indonesia^[c]^, Australia (2x)

Participants were selected from an online survey among providers of PrEP for HIV prevention; six additional non-survey participants were recruited as identified by technical staff of the World Health Organization (WHO) Global HIV, Hepatitis and Sexually Transmitted Infections Programmes. Numbers indicate total participants per region and by PrEP provider type. Numbers in brackets after countries indicate multiple participants from a country.

^[a]^ Regions refer to the WHO regions.

^[b]^ Included one pharmacist, one researcher, one PrEP navigator, one counsellor, and one clinical psychologist.

^[c]^ Providers interviewed who did not participate in online survey

### Differentiated Service Delivery

Across regions, PrEP providers supported differentiated service delivery and recommended expanding these approaches to increase uptake and persistence on PrEP. Adapting services to the needs and preferences of people who could benefit from PrEP was viewed by PrEP providers as important to improve acceptability, maintain services during COVID-19 restrictions, and to ensure access for priority populations that may face access challenges.

#### Where Should PrEP Services be Delivered?

PrEP providers reported using a range of community-based service delivery models, including clinics managed by community-based non-governmental organizations, mobile and home-based services, and telehealth (such as the use of online and text messaging systems) linked with local laboratories and pharmacies. In some settings, clients face considerable travel time and costs to reach services, experience stigma and criminalization, or have limited mobility due to COVID-19 restrictions or political unrest. Providers felt that community-based services improve access to PrEP for these clients and reach those who may be reluctant or unable to seek facility-based services. Moreover, providers noted that community-based services may reach individuals who may not recognize or acknowledge their risk and that they could benefit from PrEP, especially young and marginalized groups. One provider of services for adolescent girls and young women highlighted the importance of outreach through peer educators in communities and schools to encourage this population to understand their risk and the benefits of PrEP.

*“Our clients often do not recognize that they are at risk, and so we often take services to them [in their communities].” (Nurse, Namibia)*

During the COVID-19 pandemic, telehealth and mobile services ensured that people could continue accessing PrEP. In some settings, PrEP was integrated into other vital services such as opioid replacement therapy and outreach systems including for unhoused populations. Providers in high-income settings in particular noted that COVID-related adaptations have become standard in many settings as they found clients were more likely to effectively use PrEP when there are fewer requirements to visit clinics. In contrast, some providers from lower-income settings noted that workforce limitations and supply constraints limit the ability of services to continue offering PrEP services outside of healthcare facilities.

*“[...] another COVID-19 change [in PrEP service delivery that has been maintained] is that now the STI samples are collected at home – a safer and [more] comfortable way for people [to] spend less time at the facilities.” (Nurse, Spain)*

Alternative service delivery channels were also seen as reducing pressure on clinic staff, allowing them to allocate more time to clients with complex needs.

*“One of the barriers in high-volume services is limited staffing. In this case, self-sampling [for HIV testing] is instrumental to [meeting the demand for] PrEP services; clinical decision-making is done in a virtual space, and prescriptions are sent by post. But there’s a minimum level of interaction required by law, for medical safety.” (Physician, UK)*

Providers also emphasized the benefits of providing PrEP in a range of routine settings due to stigma associated with specialized clinics and HIV services.

*“We are not delivering PrEP through specialized HIV services as [clients] are afraid of stigma. So, this is reducing a significant barrier – they go to regular family health clinics in the private sector for PrEP services.” (Physician, Guatemala)*

Telehealth models were commonly described to be linked to local laboratories and pharmacies for HIV testing and PrEP supply, increasing access in settings where there are few clinics providing PrEP. Some providers used online services to send drugs directly to clients after confirming HIV test results. Providers across regions noted that telehealth models are used for PrEP continuation – not initiation – mainly for clients who were seen as adherent and stable on PrEP.

*“Telemedicine and new ways of communicating [via WhatsApp] were implemented during the last 2 years. [We] also send medication for those who cannot go easily to the center, after one year of [effective] PrEP [use].” (Nurse, Spain)*

HIVST was considered integral to community outreach and providing PrEP services outside of clinic settings, including telehealth services, especially during COVID-19 restrictions. Providers from all regions reported how HIVST helps to reach vulnerable populations that face stigma or criminalization and provides linkages to PrEP services.

“*There is a big cohort of very closeted and fearful, culturally and linguistically isolated people who we haven’t been able to reach [...] and they would benefit from self-tests [for earlier engagement in care].” (Nurse, Australia)*

In settings with limited laboratory resources or where stigma is a deterrent to seeking HIV testing services, HIVST can reach people who may benefit from PrEP or HIV treatment services.

*“For some people, [HIVST] can lead to the first opportunity to engage with any kind of HIV service.” (Physician, Guatemala)*

Most providers felt that HIVST is a useful tool as long as users understand its limitations, how to use it, and how to access follow-up counselling and care. However, providers generally felt that HIVST should not be used for PrEP initiation due to beliefs and perceptions that it could miss acute HIV infections. One provider unconditionally supported using HIVST for PrEP initiation; in that setting, PrEP is offered only in one urban area, and the provider believed that self-testing could increase access to PrEP.

*“PrEP is only provided in Yangon, and so people outside of the city who want PrEP need other options. It would be better if there was a recommendation for HIVST for initiation so that we could use this for virtual services.” (Physician, Myanmar)*

One provider in the US felt that “2-step testing” – using a self-test result for immediate initiation with a confirmatory test within one week of initiation – may be acceptable in some situations. The provider noted that benefits of HIVST may outweigh any perceived risks about missing an acute HIV infection.

*“The benefit of PrEP for an individual at significant risk of HIV may outweigh the risk of initiating that person on PrEP with an acute HIV infection.” (Pharmacist/clinician/researcher, USA)*

In addition to concerns about missing acute HIV infection when using HIVST, some providers raised concerns that, when HIVST is used to deliver PrEP outside of clinics, there may be fewer opportunities for direct engagement with clients to assess health status and to discuss sexual health issues and the benefits of using PrEP than when clients use clinic-based services.

*“[The initiation visit] is an opportunity to have a full panel of labs.” (Physician, Lebanon)*

*“It doesn’t matter if it’s a self-test or a [provider administered rapid diagnostic test], and they are both non-reactive [...]; the issue is that we still need to convince [...] people that they are at risk, and that they need PrEP.” (Physician, Nepal)*

Most providers viewed HIVST as useful for continuation, and several providers recommended this approach to support differentiated services delivery of PrEP and reduce frequency of clinic visits.

*“We advise people to come in for a lab test every 6 months, with a self-test used every 3 months [for refills] if they want to do that.” (Physician, Thailand)*

#### When Should PrEP be Delivered?

While emphasizing the need for national and international guidance, offering flexibility and simplified service delivery was sometimes mentioned as more important than strictly following available guidance when delivering person-centred PrEP services. Some providers reported deviating from national guidelines to improve uptake and effective use of PrEP in their settings. For example, when a client who is known to have been using PrEP effectively cannot come to the clinic, one provider in the US suggested that three-monthly HIV testing for PrEP refills can be waived to ensure continued protection. One provider in Europe noted that they have already reduced clinic visits to every six months as demand has increased but clinic staffing and budgets have remained static; clients still must do an HIV test every three months for refills but can do this at a laboratory convenient to them. In some settings, multi-month dispensing was considered preferable to risking an interruption in PrEP. Some providers felt that six-monthly testing and dispensing would be preferable to the current three-monthly schedule for client convenience as well as alleviating pressure on staff. However, several providers noted that national guidelines often constrain this type of flexibility.

*“I want to be in line with the guidance and I also want to be in line with what my clients need.” (Physician, Thailand)*

Some providers also raised the issue of flexibility in terms of testing requirements, emphasizing the occasional need to allow PrEP initiation or dispensing prior to laboratory results being available, and operational considerations.

*“We work flexible hours; it is very easy to change appointments and we do not punish our clients who do not comply. We basically follow the national guidelines, but we offer PrEP to all those who request it (without medical contraindications), and after a year of taking PrEP, we alternate face-to-face visits with visits where PrEP users self-test [...]. We can send PrEP by courier for those who live far away.” (Nurse, Spain)*

#### What Should be Provided?

Several providers felt that PrEP should not be offered in isolation, and that offering more comprehensive services could significantly increase acceptability and thus increase PrEP uptake and persistence. Providers indicated a particular need for services addressing mental health, gender-affirmation, harm reduction, chemsex, and sexual and reproductive health.

*“We would love to be able to do all of those services in-house because if you can fold in gender-affirming hormone therapy with HIV prevention, in a sensitive, inclusive space [...] it just makes sense.” (Nurse, Australia)*

*“When there is not a comprehensive package of services that are designed to meet their needs, some people will choose not to seek care.” (Physician, Viet Nam)*

#### Who Should Provide PrEP?

In most upper middle- and high-income countries, providers reported that physicians were mainly responsible for prescribing PrEP for initiation and continuation, while nurses and other cadres, such as PrEP navigators and pharmacists, support assessments, counselling, testing, dispensing, or clinical monitoring. In some cases, those providers noted that nurses and pharmacists are also allowed to prescribe PrEP. PrEP providers from the Africa region described that prescription by non-physicians – mainly nurses and some pharmacists – is more common. Most providers interviewed felt that other healthcare cadres, as well as lay and peer providers, should be involved in PrEP provision, including prescription, although they emphasized the important clinical role of physicians for complex cases. Task sharing was generally considered necessary to meet the increasing demand for PrEP and to make PrEP services more acceptable and sensitive to the challenges faced by many PrEP clients. Some interview participants noted that PrEP providers themselves may create barriers to accessing services, for instance due to lack of awareness about PrEP, unwillingness to prescribe PrEP, and stigmatizing attitudes toward clients who may benefit from PrEP. Providers noted that task sharing, particularly with peers, may overcome some of these barriers, but the importance of training to provide non-judgmental services for all providers was emphasized.

*“With PrEP you need to be aware of the person in front of you – their lifestyle, their drug addictions, etc. As long as the person knows what they are doing and knows the community they are serving, [I have] no problem with other providers.” (Physician, Switzerland)*

*“The resources that a PrEP navigator with lived experience can provide are invaluable to helping people be fully informed and motivated to stay on PrEP for as long as they need it.” (PrEP navigator, USA)*

### New PrEP Products

Study participants noted that new PrEP products – the DVR and CAB-LA – have the potential to engage more clients in PrEP services who find it difficult to use pills or prefer more discreet options. However, various concerns were raised, including regarding costs and side effects and, for CAB-LA, risks of HIV drug resistance and questions around HIV testing requirements. Table 2 lists key perceived benefits and concerns raised by PrEP providers regarding CAB-LA and the DVR.

**Table 2:** Perceived benefits and concerns regarding long-acting injectable cabotegravir (CAB-LA) and the dapivirine ring (DVR) as pre-exposure prophylaxis (PrEP) by participants

	Perceived benefits	Concerns
**CAB-LA**	• Reduces need for adherence to pill taking • Less reliance on client’s ability to assess risk for HIV acquisition • Discretion	• Side effects and interactions with other drugs • HIV testing requirements • Costs • Risks of HIV drug resistance, particularly after discontinuation • Increased pressure on providers due to more complicated and time-consuming administration and reliance on physicians for injections • Difficulty of delivery in non-clinic settings and remedicalization of PrEP • Frequency of clinic visits may be burdensome for some clients
**DVR**	• Reduces need for adherence to pill taking • Self-managed care and minimized need for clinic visits • Discretion • Minimal systemic absorption of drug • Potential to engage more cisgender women in PrEP services, especially younger women and female sex workers	• Not as effective as oral PrEP or CAB-LA • Insertion and removal may be challenging for some clients • Costs • Acceptability in some countries • Misunderstandings regarding contraception • Potential for vaginal infections in some settings with poor hygiene and sanitation

CAB-LA: Long-acting injectable cabotegravir; DVR: dapivirine vaginal ring; PrEP: pre-exposure prophylaxis

*“New technologies take time to adopt. It’s up to PrEP providers to educate people and communicate effectively about new products.” (Physician, Thailand)*

#### CAB-LA

PrEP providers expressed excitement around CAB-LA as an additional PrEP option, noting that removal of the need to adhere to pill taking, which can be a significant challenge for clients, and the increased discretion of bi-monthly injections would be important drivers of demand for CAB-LA.

*“A lot of people are excited about injectable PrEP because they don’t have to worry about adherence, and there will be more privacy.” (Physician, Viet Nam)*

For some populations, particularly young people and individuals with unstable lives, CAB-LA represents an easier option that depends less on adherence and risk perception.

*“It is easier to convince someone to take an injection than to expect adherence [to a daily pill].” (Nurse, Namibia)*

*“Some clients struggle with risk assessment, and they don’t understand the pharmacokinetics, [so it’s] difficult to convince them that daily PrEP is the best protection for them; in cases like these, the injection will be much easier for the provider as well as the client.” (Nurse, USA)*

*“It is difficult to maintain the motivation to adhere to a daily pill without an HIV diagnosis.” (Physician, Mexico)*

Client enthusiasm for an injectable and long-acting option was considered an important facilitator for uptake of PrEP, and several providers noted that clients were already asking for this product. Some providers noted that many people already have experience with injectables for other healthcare needs.

*“Cabotegravir is a novel concept within PrEP, but it’s not a novel concept in general [referring to injectable contraception and injectable medications for a range of health issues], but it’s a creative way to help prevent new cases of HIV transmission.” (Pharmacist/clinician/researcher, USA)*.

Personal preferences were considered an important determinant of PrEP uptake and providers emphasized the need to preserve choice.

*“Some people are afraid of injections, so you have to keep both options available.” (Physician, Lebanon)*

*“I definitely don’t see [CAB-LA] as the answer to all the problems, but it’s a very useful new means of prophylaxis.” (Physician, Poland)*

Most providers felt that CAB-LA would be difficult to deliver in non-clinic settings due to a reliance on physicians or nurses to administer injections and to review complex health issues or drug interactions. Reasons for this included legal restrictions and limited capacities of alternative service delivery sites (e.g., pharmacies, which may have limited space and time for CAB-LA provision) in some countries and cultural factors in others. Some providers noted risks that an injectable form of PrEP may remedicalize PrEP, reversing some of the benefits of differentiated service delivery. In some settings with limited staffing, providers were concerned that the demand for long-acting PrEP will exceed the availability of qualified providers to deliver it.

“*We have no staff to provide injections outside the facility.” (Physician, Zambia)*.

However, most providers felt that non-physician cadres could be trained to administer injections – reflecting on experience with COVID-19 vaccination campaigns that involved different cadres – if national laws allow it and physicians are available for consultations and complicated cases. In some cases, the notion of self-injection was considered feasible.

Given uncertainty about the costs of CAB-LA, some providers suggested narrow targeting to ensure the best use of resources. Some would prioritize clients whose lives are unstable, making daily adherence challenging; other providers, noting concerns about adherence to injection schedules, proposed offering CAB-LA only to clients who have demonstrated adherence and effective use of daily oral PrEP to avoid “wasting” resources.

Many providers expressed concerns about HIV drug resistance due to the long pharmacokinetic tail of CAB-LA after discontinuation. This was particularly noted by providers in low- and middle-income countries where service and supply chain disruptions are common. PrEP providers also noted that CAB-LA users who choose to stop may find it especially challenging to commit to alternative HIV prevention. One provider expressed concern about the risk of resistance as clients may discontinue due to frequent, uncomfortable injections.

*“While it sounds really attractive, in practice it requires rigorous adherence to a more frequent clinic schedule than oral PrEP, and the intramuscular injections are not pleasant.” (Physician, UK)*

Several providers expressed concern about the need for nucleic acid technology (NAT) testing to reduce the possibility of initiating a client on CAB-LA with acute HIV infection. PrEP providers, particularly those outside of high-income countries, noted that NAT is neither widely available nor affordable, and limited laboratory capacity is needed for HIV treatment services.

*“It could be difficult to ask [government labs] to do extra viral load testing for people who are HIV-negative. We’re still struggling to get viral load testing for people on antiretroviral therapy [...] due to shortages of reagents, machine breakdowns, transportation problems.” (Physician, Nepal)*

*“It depends on the attitude of the chief physician. For now, lab services for PCR testing are reserved only for HIV-positive patients. PrEP is [...] not a priority.” (Physician, Ukraine)*

*“Each country will have to decide if they will offer CAB-LA depending on their health system and whether or not they can deliver CAB-LA safely with the required testing to make sure that clients do not have an acute infection.” (Researcher/clinician, Brazil)*

#### DVR

Many providers interviewed serve populations of men who have sex with men and transgender women and consequently had limited views on the DVR. However, most providers who were familiar with it felt that DVR would be very useful for cisgender women as it provides an additional choice for HIV prevention, and it offers benefits in terms of avoiding adherence to a daily regimen, self-management, and less systemic absorption compared to other PrEP products. A few providers noted that DVR could be a way to get more cisgender women engaged with PrEP services, while noting that there may be challenges with introducing a new prevention product.

*“This is going to be hugely important – a major game changer – especially for young girls. If they could link it with contraception, it would be amazing. Women are not always able to communicate their wants and needs, and this will give them more discretion and autonomy and hopefully engage more women into PrEP.” (Nurse, USA)*

*“I think our clients will really appreciate the privacy of the vaginal ring, but it may take some time for providers to help them learn how to use it.” (Nurse, Namibia)*

One provider in Latin America noted that, while the DVR was not used in the region, it would be an excellent option for female sex workers. However, general barriers for accessing PrEP remain for this population as they experience significant stigma in health services and would be reluctant to disclose their reason for seeking PrEP. Similarly, several providers noted that stigma around sexuality and self-managed sexual health products may create barriers for cisgender women. Providers in the South-East Asia region noted similar challenges with the female condom and contraceptive rings.

*“It sounds interesting, but Asian women are not comfortable to talk about sex or using products that require self-management. [It will] probably need a lot of demand generation [as there are cultural] barriers for Asian women to use these products.” (Physician, Viet Nam)*

Some providers raised questions around the efficacy of the DVR compared to oral PrEP.

*“It would be a good option for female sex workers, adherence is a challenge for them, so the ring would help. But female sex workers are also very familiar with PrEP, and they would prefer this to the ring as it is more effective.” (Physician, Thailand)*

Other concerns raised by providers included costs; poor commodity management in some settings that will affect dependability of supplies; and potential additional pressures on staff to provide instruction on insertion and removal as well as clear information about the DVR having no contraceptive effect. One PrEP provider from the Africa region raised concerns about potential bacterial infections and unknown effects on fertility.

## Discussion

The results of our qualitative consultations with PrEP providers globally highlighted a range of ways in which providers have been delivering PrEP, adapting services to meet the needs of their clients, and successfully addressing the challenges of delivering services during COVID-19. New PrEP products, offering long-acting and more discreet protection, have the potential to improve use of PrEP further, and providers have important views on the potential benefits and concerns about these new HIV prevention options. These insights can contribute to developing guidance that will drive continued progress on HIV prevention and identify what providers need to support effective programme implementation.

PrEP providers across countries broadly agreed on the benefits of differentiated PrEP service delivery and reported particularly positive experiences with community-based approaches, including the use of telehealth, to maintain services during COVID-19. A study in the US found similarly strong support for telehealth models for PrEP delivery, although providers in that study emphasized the need to maintain access to alternatives to telehealth, such as community-based PrEP provision and specimen self-collection, as telehealth may not be suitable for all clients [26]. This aligns with preferences for easily accessible services expressed by many PrEP clients [14]. While providers pointed out that the benefits of providing services outside of facilities can ensure more equitable access, particularly for marginalized or criminalized populations, providers also emphasized the benefits of facility-based services, including for clinical assessments and referrals for clients who may require more intensive support. Some clients may also prefer accessing PrEP services in health facilities, so it is important that programmes offer a range of service delivery points, both in facilities and outside, so that clients can choose where they want to access PrEP services.

HIVST was considered useful to enable many of these community-based approaches and was particularly valued by providers in low- and middle-income countries. However, while providers had favourable views on using HIVST for PrEP continuation, there were concerns about using HIVST for initiation. Recent WHO guidance suggests that HIVST represents an additional approach for initiation, continuation and re-initiation of oral PrEP and the DVR [5]. It is increasingly recognized that people commonly start, stop and re-start oral PrEP [27]; HIVST may be particularly useful to support these patterns of PrEP use. Where programmes consider HIVST for PrEP, close engagement with and training for PrEP providers is critical to address any reservations, misconceptions, or lack of implementation experience they may have. Several PrEP providers in our consultations noted concerns about missing acute HIV infections when using HIVST. However, a modelling study of significant scale-up of oral PrEP in Kenya, with about 45 million PrEP initiations over 20 years, found that the proportion of individuals inappropriately initiated on PrEP while acutely infected with HIV was < 0.01% for HIVST [28]. While the proportion of inappropriately initiated individuals using provider-administered rapid diagnostic tests was about three-quarters lower – and using NAT nearly eliminated the risk – these risks were so small that PrEP use in none of the testing scenarios significantly contributed to population-level drug resistance. This suggest that the benefits of choosing an HIV testing approach that enables differentiated service delivery approaches and thus contributes to PrEP uptake and use, such as the use of HIVST, likely outweigh the small risks (although WHO guidance notes that considerations are different for CAB-LA [5]).

As identified by providers in our interviews, lack of awareness, willingness to prescribe PrEP and confidence as well as biases and stigmatizing attitudes among PrEP providers can represent barriers to PrEP access. This has been similarly found by other studies [17–24], underlining the need for training of providers to provide non-judgmental services, although previous research tended to focus on the US. Providers in our consultations suggested that task sharing with a range of healthcare cadres and lay and peer providers can provide less stigmatizing, more acceptable services and reduce pressure on the health system. This was similarly found in a study in Zimbabwe which noted the benefits of nurses for providing PrEP services for adolescent girls and young women [24]. A study in Thailand also found that closer collaboration between hospital-based PrEP providers and key population-led health services can reach more potential PrEP users with sensitive and effective services [19].

PrEP providers across countries were generally enthusiastic about the prospect of new long-acting, injectable PrEP. Studies among current or potential PrEP users similarly found interest in and preference for injectable PrEP, although there is considerable variation in preferences across populations and regions [15]. However, providers noted concerns, particularly regarding costs, feasibility of HIV testing requirements, and drug resistance. These concerns are in line with evidence from clinical trials that found that HIV drug resistance can emerge where individuals are initiated on CAB-LA while already infected with HIV or when becoming infected after exposure to CAB-LA [29]. While a limited number of cases of drug resistance could possibly have been prevented by the use NAT testing [30], providers in our interviews noted that NAT was not routinely available in their settings. Unavailability of NAT, as well as lack of regulatory approval for diagnosis of HIV in adults, lengthy turnaround times, and high costs limit the feasibility of NAT testing [13], and WHO guidelines state that national HIV testing algorithms can be used for CAB-LA [12]. PrEP providers repeatedly raised concerns around drug resistance, particularly after discontinuation of CAB-LA, although this has not been observed in clinical trials [31]. Moreover, mathematical modelling suggests that population-level benefits of CAB-LA in terms of reduced HIV incidence likely outweigh harms from drug resistance [32]. It should be noted that few interview participants had experience with provision of this new option; their concerns and misperceptions need to be addressed through training and support for PrEP providers to ensure effective implementation of CAB-LA.

Providers, particularly from less well-resourced settings, also expressed concerns about human resource capacity to provide an injection-based intervention that required relatively frequent clinic visits. While task sharing can facilitate the delivery of PrEP services [10], participants in our consultations raised concerns about regulatory restrictions regarding who can administer intramuscular injections, and management of possible side effects or any drug-drug interactions. These issues are related to broader concerns about how the integration of CAB-LA could lead to a remedicalization of PrEP services – requiring more clinic visits and staff who can administer injections – and undermine some benefits of community-based PrEP services, especially for stigmatized populations. As programmes consider the introduction of CAB-LA and implementation projects are developed, the risks of remedicalization of PrEP services should be considered and, where possible, differentiated service delivery approaches should be evaluated, such as delivery in community and pharmacy settings and using a range of providers to deliver PrEP and other services.

Most PrEP providers had fewer concerns about implementation of the DVR. One common concern included the additional time for training cisgender women on insertion and removal of the ring, and potential bacterial infections in some settings where poor sanitation infrastructure is common. In some settings generating demand for the ring was perceived as a challenge due to cultural norms, and it has not been approved for use in some countries. But given the limited systemic absorption of the DVR, low concern about drug resistance and the lack of impact on HIV test performance, the DVR could be an important self-care product available in community settings, including pharmacies, and supported by HIVST. Many providers felt that for adolescent girls and young women, female sex workers, and other women who face barriers to attending services, or who prefer more discreet options, the DVR will be an important additional choice for HIV prevention, although some had questions about the efficacy of this product. When used consistently, the efficacy of the DVR is likely higher than initially reported [33], and some studies suggest that some women prefer the ring when given a choice between the DVR and oral PrEP [34]. However, due to the suboptimal efficacy, it is important that the DVR is always offered together with other PrEP options and clients are provided with information to make informed decisions regarding which PrEP product best suits their needs and preferences. Implementation projects are planned that will offer the DVR together with both oral PrEP and CAB-LA [35], which will provide insights into how such informed decision making can be supported by providers. Training of providers will be critical as providers also expressed some misunderstandings regarding the DVR in terms of drug-drug interactions or possible effects on fertility.

While interview participants in our consultations were selected from a random sub-sample of a large online survey, they were selected based on their knowledge of key topic areas and to reflect geographic representation, gender balance, and a mix of professional cadres. Selection criteria included experience with community-based delivery and knowledge of new PrEP products, so their views may not be representative of PrEP providers more widely. It is also likely that the experience and views of such a small sample of providers do not represent the wide-ranging realities within and across regions.

Despite these limitations, our consultations included PrEP providers from all regions, and they revealed important insights into providers’ views on PrEP service delivery. Many of the findings, which had not been generated in other, narrower studies on PrEP, were used to inform recent WHO guidance on differentiated services and CAB-LA; they identify opportunities for expanding PrEP and indicate ways to address providers’ concerns through future research, guidance and support for capacity building.

## Conclusions

Provider perspectives are critical for the development of guidelines and programmes that make PrEP more accessible and acceptable. Our consultations suggest that providers are supportive of expanding access to PrEP through differentiated service delivery, and they view the emergence of new products as potentially instrumental in creating demand for PrEP services and supporting effective use of PrEP. Providers also signal the challenges of delivering PrEP in settings where access to services is constrained by a range of barriers related to resources, commodity management systems, stigma, and, in some cases, political will. There is also a lack of awareness about PrEP in some settings, including misconceptions. Understanding areas where provider capacities and biases may create barriers for their clients can help to define the training, information and support needed to ensure that providers can deliver effective programmes, providing more acceptable services, and improving uptake and use of PrEP.

### Electronic Supplementary Material

Below is the link to the electronic supplementary material.

Supplementary Material 1

## Data Availability

The interview guide is included here as a Supplementary file. The data generated and analyzed are not publicly available as no explicit consent was obtained from participants to make all data publicly available. Participants only consented to make specific quotes and general results from their interviews available through publication. Data are available from the corresponding author on reasonable request.
