# Supplementary material for: The Future of Pre-Exposure Prophylaxis (PrEP) for HIV Prevention: A Global Qualitative Consultation on Provider Perspectives on New Products and Differentiated Service Delivery
Source: AIDS Behav. 2023 Jun 23;27(11):3755–66. doi: 10.1007/s10461-023-04093-1 (PMC10589125; doi:10.1007/s10461-023-04093-1)
Supplement: Supplementary file 1 — Supplementary Material 1 [file 10461_2023_4093_MOESM1_ESM.pdf]

The future of pre-exposure prophylaxis (PrEP) for HIV prevention: Provider perspectives

Henderson et al.

Supplementary material: Semi-structured interview guide

This supplementary material provides the semi-structured interview guide that was used for the in-depth interviews conducted with PrEP providers. This generic guide was adapted to each interview participant.

Semi-structured interview guide

INTRODUCTION

Thank you for making time to participate in this interview.

The main objectives of this interview are to:
1) learn your **views on new PrEP products** that are becoming available;
2) **follow up** on some of the issues around differentiated service delivery that were raised in the PrEP providers survey.

Consent

Do you consent to participate in this interview about your work as a PrEP provider? I would like to record our conversation for analysis purposes; if you prefer not to be recorded I will just take notes.

1. New products

CAB-LA and DVR

- I see that you do/do not have experience providing the PrEP ring / CAB-LA as part of a trial or study in your setting. When it becomes available, do you think it will it be useful in your setting? Why? Why not?
- What do you anticipate will be the main challenges with this product? Likely acceptability?
- What will be needed to generate demand for this product?
- Do you think your current PrEP service delivery practice needs to be adapted to provide this product? If so, in what way?
- Do you see any challenges with your current HIV testing algorithm to implement CAB-LA? What changes do you think are necessary (probe: viral load / NAT testing)? If you use HIVST in your service, do you think it could be used in the context of CAB? (Link it to the way HIV testing currently done, do they foresee any issues with that algorithm when it comes to CAB? Probe on viral load/NAT and HIVST)

>>> Feasibility prompts could include:

1) Is this product suitable for delivery outside of traditional healthcare settings (e.g., mobile service provision)?
2) Who would be able to deliver the product (e.g., nurses, others)?

1. Barriers to uptake and delivery, and adaptations (differentiation) to minimize these

- Are there barriers (for users and/or providers) to PrEP uptake and continuation in your service?
- If yes, what are the main barriers? Are they related to national guidance or other issues?

- Have you done anything to **make it easier** for clients to use services or to **adapt services** to their needs? Are adaptations in line with national guidance? (Were these adaptations necessary during to C19?)

>>> For example, do you offer: (only if respondent needs a prompt)

- **Same-day PrEP initiation** (coming to get PrEP at a site, rapid initiation on PrEP, leave with PrEP drug supply)
- **HIV self-testing**
- **PrEP outside of traditional healthcare settings**? Describe… advantages/disadvantages (possible prompt: Could CAB-LA be provided in this service delivery model outside of healthcare settings?)
- **Multi-month dispensing**: Do you give supplies of PrEP for 3 or more months?
- **HCV self-testing**
- **STI self-sampling?** (better uptake/acceptability than sampling different anatomical sites in clinics?)

>>> Ask about advantages and disadvantages of those efforts to simplify/differentiate

- If no changes have been made, are there ways that you would like to see PrEP services adapted in the future in order to remove or minimize barriers? Are these changes possible given current guidelines or would the guidelines need to change?

>>> Explore the challenges of differentiated services.

- In your practice, do you feel it's more important to <u>simplify</u> PrEP (making it easier/quicker to get more people on PrEP) or <u>adapting</u> services to make them more tailored to individuals' needs? Or equally impt?
- What do you see as the challenges involved in differentiation?

Pre-initiation testing

- Do you think pre-initiation testing requirements should be minimized or simplified? Ways to do this?

>>> probe re HIVST – is this appropriate and useful for initiating people on PrEP? Does it make services easier to deliver or harder? Advantages? Concerns?

Continuation testing

- Do you think testing requirements for continuation should be minimized or simplified? Ways to do this?

>>> probe re HIVST – is this appropriate and useful to replace clinic visits for continuation? Advantages? Concerns?

More on HIVST

- In general, how do you view HIVST – advantages/concerns? Increased costs? Increased time?
- Do you feel that HIVST supports PrEP service delivery? If yes, how?
- <u>As appropriate: I see that you said</u> that pharmacy-based delivery is available in your setting -- Is HIVST used for these services?

>>> Note: Distinguish between hypothetical vs real-world experience

2. Differentiated service delivery

What should be delivered?

- On the survey, you listed a number of services offered to PrEP clients at your service. Are there any other services you think people attending for PrEP initiation or continuation should be offered that are not currently offered at your service?
- Do clients ask for other services? What are they?

Where / how to deliver PrEP services?

>>> For those who have experience with c-b delivery:

- Do you think community-based service delivery (outside traditional health-care settings) is useful for increasing PrEP uptake and continuation (regardless of whether you have experience with this or not)?

If prompt needed:
  - Fixed community site (e.g. NGO)
  - Mobile community site (e.g. mobile van)
  - Telehealth
  - Pharmacy (health-care setting / fixed community site)

- In your experience, what are the advantages of community-based delivery?
- What are the challenges with community-based service delivery?
- What is the biggest barrier to community-based delivery?

>>> If the respondent has experience with pharmacy-based delivery:
- Are there any challenges with pharm-based delivery?
- What is the biggest barrier to pharmacy-based delivery?

Who can/should deliver PrEP?

>>> I see that you responded that in your service XX can prescribe PrEP for initiation/continuation...
- Do you think there are providers in the health workforce who are not currently able to prescribe PrEP who could be trained to do this? E.g. pharmacists, nurses, peer/lay providers....
- Apart from prescribing PrEP, do you think it would be beneficial if other healthcare cadres were more involved in different aspects of PrEP service provision? E.g. pharmacists involved in eligibility assessment...

4. Other implementation issues

- Are there any other issues you would like to raise about PrEP implementation, simplification and differentiated service delivery for increased uptake, improved adherence, etc?
